# Assessment of the Diversity of Fungal Community Composition Associated With *Vachellia pachyceras* and Its Rhizosphere Soil From Kuwait Desert

**DOI:** 10.3389/fmicb.2019.00063

**Published:** 2019-01-31

**Authors:** Majda K. Suleiman, Kingsley Dixon, Lucy Commander, Paul Nevill, Ali M. Quoreshi, Narayana R. Bhat, Anitha J. Manuvel, Mini T. Sivadasan

**Affiliations:** ^1^Environment and Life Sciences Research Center, Desert Agriculture and Ecosystems Program, Kuwait Institute for Scientific Research, Kuwait City, Kuwait; ^2^ARC Centre for Mine Site Restoration, Department of Environment and Agriculture, Curtin University, Perth, WA, Australia; ^3^School of Biological Sciences, The University of Western Australia, Perth, WA, Australia

**Keywords:** soil fungal communities, Lonely Tree, *Vachellia pachyceras*, rhizosphere soils, Kuwait desert ecosystem

## Abstract

This research examined the general soil fungi and AM fungal communities associated with a Lonely Tree species (*Vachellia pachyceras*) existing in the Sabah Al-Ahmad Natural Reserve located at the Kuwait desert. The goals of the study were to describe the general fungal and AM fungal communities present in the rhizospheric, non-rhizospheric soils and roots of *V. pachyceras*, respectively, as well as local and non-local *V. pachyceras* seedlings when grown under standard nursery growing environments. Soil and root samples were analyzed for an array of characteristics including soil physicochemical composition, and culture-independent method termed PCR-cloning, intermediate variable region of rDNA, the large subunit (LSU) and internal transcribed spacer (ITS) region sequence identifications. The results reveal that the fungal phylotypes were classified in four major fungal phyla namely Ascomycota, Basidiomycota, Chytridiomycota, and Zygomycota. The largest assemblage of fungal analyses showed communities dominated by members of the phylum Ascomycota. The assays also revealed a wealth of incertae sedis fungi, mostly affiliated to uncultured fungi from diverse environmental conditions. Striking difference between rhizosphere and bulk soils communities, with more fungal diversities and Operational Taxonomic Units (OTUs) richness associated with both the field and nursery rhizosphere soils. In contrast, a less diverse fungal community was found in the bulk soil samples. The characterization of AM fungi from the root system demonstrated that the most abundant and diversified group belongs to the family Glomeraceae, with the common genus *Rhizophagus* (5 phylotypes) and another unclassified taxonomic group (5 phylotypes). Despite the harsh climate that prevails in the Kuwait desert, studied roots displayed the existence of considerable number of AM fungal biota. The present work thus provides a baseline of the fungal and mycorrhizal community associated with rhizosphere and non-rhizosphere soils and roots of only surviving *V. pachyceras* tree from the Kuwaiti desert and seedlings under nursery growing environments.

## Introduction

Kuwait is located in the northeastern corner of the Arabian Desert where it constitutes a part of the northwestern coastal flat of the Arabian Gulf. This arid region severely suffered in recent years from prolonged drought (Khalaf, 1989; Brown and Porembski, 1997; El-Sheikh et al., 2010). This has ultimately steered a decline in soil and plant productivity (El-Sheikh et al., 2010), leading to further desertification. Furthermore, increases in anthropogenic activities have exacerbated these processes and under incessant risk of desertification (Wang et al., 2006). Consequently, about 90% of Kuwait’s territory (an area of approximately 18,000 km^2^) is occupied by the desert (Kaitharan, 2013). Such a degraded soil situation needs to be reversed to its original state to conserve key native plant species and this may include a series of interventions aimed to sustain soil stability and productivity.

In Kuwait, *Acacia gerrardii* (Syn *Vachellia gerrardii)* is considered the only native tree species existing in the desert ecosystem (Boulos and Al-Dosari, 1994). *A. pachyceras* O. Schwartz, synonym *A. gerrardii* Benth., subsequently referred to as *A. gerrardii*, commonly known as “Lonely Tree (LT).” *A. gerrardii* is considered the only native tree species existing in the Kuwait desert ecosystem (Boulos and Al-Dosari, 1994) and is only available in Sabah Al-Ahmad Natural Reserve (a 320 km^2^ protected area formerly acclaimed as Kuwait’s first National Park) where it is as key-stone species (Boulos and Al-Dosari, 1994; Kaitharan, 2013). It belongs to the *Acacia* genus, one of the largest genera of leguminous tree and shrubs that has a wide distribution throughout the world (Pedley, 1986; Sene and Sylla, 2014). Recently this iconic tree is genetically identified as *V. pachyceras* based on multi-locus plastid gene sequences (Suleiman et al., 2018). Many members of this genus are recognized as species that are useful for re-vegetation of water-stressed and low-nutrient soil environments (Sene et al., 2012b; Sene and Sylla, 2014). *V. pachyceras* was, however, damaged by the gulf war in 1990 and, the remaining plants patchiness are under the threat of extinction (Kaitharan, 2013). Conservation efforts are therefore required to make this species’ survival in Kuwait’s terrestrial ecosystem and ensuring sustainability and preserving soil biological attributes.

There is increasing evidence that trees can play a key role in ecosystem rehabilitation or restoration (Donfack et al., 1995; Vincke et al., 2010; Sene and Sylla, 2014). Many studies have supported that the presence of trees provide a number of ecological advantages from increased soil organic matter content (Hobbie et al., 2006, 2007; Peichl et al., 2006; Rivest et al., 2009; Sene et al., 2012a, 2013; Sene and Sylla, 2014), to biodiversity conservation (Akpo et al., 2003; Grouzis and Akpo, 2006; Sene et al., 2012a; Hortal et al., 2013), and improved soil microbial activity and nutrient cycling rates (Hobbie et al., 2006; Sene et al., 2013). In addition, trees in desert regions often represent “fertility islands” for many species (Rodríguez-Zaragoza et al., 2008; Kavamura et al., 2013), which in turn influence long-term vegetation dynamics and ecosystem processes. Moreover, they have a role in combating land degradation through stabilizing soil surfaces by preventing soil erosion and in facilitating plant recruitment and survivorship (El-Sheikh et al., 2010; Sene et al., 2012b, 2013; Kavamura et al., 2013; Sene and Sylla, 2014).

Microbial associations have been pointed as an important strategy to guarantee plant growth and survival (van der Heijden et al., 1998, 2008; Sene et al., 2010) and the effect is more pronounced under arid conditions such as desert areas. Soil microorganisms are particularly known to play key roles in ecosystems, and mediate many ecological processes that are central to ecosystem functioning. These processes include nutrient acquisition (Kahindi et al., 1997; Sene et al., 2010), nitrogen cycling, carbon cycling, soil formation (Rillig and Mummey, 2006; Sene and Sylla, 2014), decomposition processes (Hobbie et al., 2006, 2007; Peichl et al., 2006), and the regulation and maintenance of plant biodiversity (van der Heijden et al., 1998, 2008; Sene and Sylla, 2014). Plants adapted to harsh environments and their associated soil microorganisms within these habitats make both partners highly competitive and adaptive (Basil et al., 2004). Highly diverse groups of fungi are represented among these soil microbial communities and play fundamental physiological and ecological roles in desert ecosystems (Meiser et al., 2014; Sene and Sylla, 2014; Powell et al., 2015). Arid region soil microbes are adapted to sustain extreme environmental conditions and maintain a leading role in ecosystem processes (Pointing and Belnap, 2012). AM fungi are one of the most important associations in terrestrial ecosystems, influencing plant productivity through the acquisition of nutrients and water (Smith and Read, 2008; Allen, 2011). They can enhance plant establishment by buffering different environmental stresses and enhancing soil properties (Jeffries and Barea, 2001). The growth and development of *A. nilotica* is improved in association with AM fungi (Reddell, 1993). Osonubi et al. (1992) reported that inoculation of AM fungi to *A. nilotica* has the capacity to increase drought tolerance and plant biomass. Although numerous studies have reported the importance of mycorrhizal symbiosis for desert tree species, the symbiotic status of this unique tree, *V. pachyceras* (LT) has never been investigated in Kuwait. Until today, no attempt has been undertaken to identify indigenous fungal or mycorrhizal populations associated with the root system of this about to extinct LT species. Therefore, it is crucial to assess the status of native fungal and mycorrhizal propagules present in the roots of surviving plants and rhizosphere soils in the Kuwait desert before undertaking revegetation programs and introducing any inoculation technologies.

The main objective of this study was to conduct a field and seedling nursery study to investigate the root systems and rhizosphere soils of the *V. pachyceras* in desert and nursery conditions. This is in addition to the examination and comparison of the rhizosphere fungal and mycorrhizal communities of local and non-local *V. pachyceras* when grown under standard nursery seedling growing conditions and media; and to evaluate plant performance related to fungal and mycorrhizal associations. The characterization of general fungal and mycorrhizal populations and functional structures were revealed using both morphological and advanced DNA-based molecular techniques. This research is the first effort in Kuwait to assess the root mycorrhizal structure of *V. pachyceras* and its rhizosphere soil fungal community composition under both desert (LT) and nursery conditions (local and non-local *V. pachyceras*).

## Materials and Methods

### Study Site and Sampling

#### Field Sampling

The experimental site was located at the Sabah Al-Ahmed Natural Reserve, Kuwait (N 29°34.909′, E047°47.734′ around the only surviving single *V. pachyceras* tree, locally known as the LT). Soil samples were collected using soil corer of 3 × 30 cm from 0 to 30 cm depth for rhizospheric soil. Lateral roots were followed through the soil excavation channels created around 80–100 cm distance from the main trunk. Each replicate sample was thus a composite of 3–4 soil samples collected, which were mixed well by placing in a zip lock plastic bag and labeled as LT-S. Additional soil samples were collected 100 m away from the LT at three different points, and served as the non-rhizospheric control soil (CTL) in subsequent analyses and it is labeled as LTCS. Roots containing root nodules were also collected in triplicates from this tree roots and labeled as LT-AM. The representative soil and root samples were collected and brought to the laboratory and stored in a refrigerator until required for analysis. Roots were cut into 1–2 cm pieces and stored in 2% cetyl trimethylammonium bromide (CTAB) at -20°C for molecular characterization of AM fungal community.

#### Nursery Sampling

Two month old seedlings of native and non-local *V. pachyceras* were transplanted into one-gallon pots containing a soil mixture of agricultural soil, peat moss, potting soil and perlite (at 2:1:1:1 ratio, v/v basis) and is named hereafter as the commercial soil mix. The commercial soil mix is used conventionally in Kuwaiti nurseries for producing large-scale nursery seedlings for the restoration program at a national scale. Therefore, the current study was also intended to investigate fungal community structures in the commercial soil mix used for growing local and non-local *V. pachyceras*. Fifteen local and non-local *V. pachyceras* seedlings each were grown in one-gallon plastic pots in the nursery, and arranged in three replicate rows with five plants in each row for duration of 1 year. Steam-sterilized commercial soil mix (SAB Potting Soil-Plantaflor) was used for this study. Non-sterilized crude commercial soil mix was used as non-rhizospheric control soil (PsBp).

Seedlings were destructively sampled for plant and soil sample collection. Soil from five pots from each replicate row was pooled together and thoroughly mixed in a plastic bag to form a single composite sample per replicate in both seedling groups. Three replicate samples were used throughout the experiment. Samples were labeled as PsApLT and PsApSA for the local (Loney Tree) and non-local (Saudi Arabia) *V. pachyceras* seedlings, respectively. Representative soil and root samples collected as described for field soil and root samples. Several root pieces were cut into 1–2 cm pieces and stored in 2% CTAB at -20°C for molecular characterization of fungal population.

### Molecular Characterization of Fungal Communities in Soil Samples

#### DNA Extraction and Amplification of ITS rDNA Region

Total community DNA from 0.25 g bulk soil and rhizosphere samples of *V. pachyceras* was extracted using a PowerSoil DNA Isolation Kit (MoBio Laboratories, Carlsbad, CA, United States) with an addition of 0.05 g of skim milk powder in the lysis buffer. The remaining steps were performed according to the manufacturer’s instructions. Isolated soil DNAs were stored at below –20°C until PCR amplification. Amplification was performed using the fungal universal primers ITS1F (5′-CTTGGTCATTTAGAGGAAGTAA-3′; Gardes and Bruns, 1993) /ITS4 (5′-TCCTCCGCTTATTGATATGC-3′; White et al., 1990).

Polymerase chain amplification (PCR) was carried out in a 25-μL reaction and consisted of 1 μL soil DNA, 1 U of *Taq* DAN polymerase (Sigma-Aldrich), 3 mM MgCl_2_, 0.2 mM dNTP mix 0.2 mg/ml of BSA, and 0.3 mM concentration of each primer. The following thermocycle program was used for amplification: 94°C for 4 min followed by 35 cycles of 94, 50, and 72°C for 60 s each, and an extension period of 72°C for 10 min using a MJ Research PTC-225 Peltier Thermal Cycler. Negative control (containing no template DNA) reactions were also conducted to assess for any experimental contamination. The PCR products, 5 μl sub-samples, were observed by electrophoresis on 1 × Tris-acetate-EDTA (TAE) agarose (1% w/v) with suitable DNA size standards (Mass Ruler^TM^, DNA Ladder Mix, Invitrogen, Canada) to ratify the size and estimate the quantity of the generated amplicons. The PCR products were visualized using ethidium bromide (0.25 μg L^-1^).

### Molecular Characterization of Arbuscular Mycorrhizal Fungal Communities in Root System

#### PCR Amplification

A nested PCR was required to obtain sufficient amplicons for the molecular characterization of AM fungal communities from the roots of *V. pachyceras* tree from the field and seedlings grown in nursery under different conditions. Before isolation of genomic DNA, roots were rinsed in sterile distilled water for 48–72 h to remove excess CTAB. Next, 50 mg of roots were bead-grinded with a Tissue Lyser II (Qiagen) using the DNeasy Plant Mini Kit (Qiagen) and following the manufacturer’s protocol. Amplification was performed using the fungal universal primers LR1- (5′-GCA TAT CAA TAA GCG GAG GA-3′) and NDL22 (5′-TGG TCC GTG TTT CAA GAC G-3′) (van Tuinen et al., 1998). The polymerase chain reaction (PCR) followed the protocol of Brito et al. (2012). The reaction was carried out in a 25-μL reaction and consisted of 1 μL of total DNA, 1 U of *Taq* DNA polymerase (Sigma-Aldrich), 1.5 mM of MgCl_2_, 0.2 mM of dNTP mix, 0.5 mM of each primer and 25 μg/μl of T4 gene 32 protein (New England Biolabs Inc.). The following thermocycle program was used for amplification: 94°C for 4 min followed by 30 cycles of 94, 56, and 72°C for 60 s each, and an extension period of 72°C for 10 min using a MJ Research PTC-225 Peltier Thermal Cycler.

The nested PCR was performed on this amplicon, previously diluted at 1:50 ratio with the primers LR1 and FLR4 (Gollotte et al., 2004). PCR conditions were almost identical to those for the first reaction; however, instead of T4 gene 32 protein, BSA at 0.2 mg/ml was added to the reaction and the annealing temperature was 55^o^C for PCR optimization. This PCR was migrated in a 1.2% agarose gel stained with ethidium bromide and visualized under UV light. Samples where amplification failed were processed once again from the first PCR.

### Cloning, Sequencing and Determination of Phylotypes and Phylogenetic Analyses

The 100 ng of PCR amplicons were cloned into a pGEM-T Easy Vector System II (Promega) using the procedure suggested by the manufacturer. The samples were stored in TTE buffer (triton X-100 1%; Tris–HCl pH 8.0 20 mM; EDTA pH 8.0 2 mM) at -20°C until use. At least 10 clones per sample were selected and transformed PCR products were sequenced using the Sanger method with two 16-capillary genetic analyzers 3130XL (Applied Biosystems).

DNA sequences were edited using BioEdit software, version 7.0.5 (^1^Hall, 1999) in order to resolve oligonucleotide ambiguities. The BLASTn (Basic Local Alignment Search Tool nucleotidic) search program algorithm^2^ was used to query the National Center for Biotechnology Information GenBank (NCBI) for highly similar sequences. When sequence similarity of at least 97% was achieved, these were considered to be in the same phylotypes (Moebius-Clune et al., 2013). Thus, one representative of each phylotype was used to continue phylogenetic analyses. Sequences were edited, aligned and queried on GenBank (NCBI) using ClustalX version 1.81 (Thompson et al., 1994) software. Operational taxonomic units (OTUs) were determined based on 97% similarity and the sequences with at least 97% similarity we considered in the same OTU, which could represent one species (Quince et al., 2008). One representative of each OTU was used to continue phylogenetic analyses. However, closely related sequences obtained were incorporated in phylogenetic analyses. Phylogenetic analyses were conducted using MEGA 6.0 (Tamura et al., 2013). Evolutionary distances were calculated as described by Jukes and Cantor (1969). Firstly, analyses were performed using the Neighbor-Joining (NJ) method (Saitou and Nei, 1987) and adopting the Kimura two-parameter method (Kimura, 1980). Secondly, maximum likelihood (ML) method based on the Kimura two-parameter model (Kimura, 1980) and 1,000 bootstrap replicates (Felsenstein, 1985) was used to compute the final tree. Finally, Bayesian inference of phylogeny was calculated using MrBayes version 3.2.2 (Ronquist et al., 2012), assuming a 4 × 4 model and non-variable substitution rates among sites – gamma rates. Analyses were constructed on two runs of four Markov chain Monte Carlo analyses where 2,000,000 generations were produced with burning fraction at 0.5 rate. These were sampled every 100 generations for 10,000 trees generated (Ronquist et al., 2012).

### Statistical Analyses

Simpson’s (*D*), Shannon–Wiener (*H*) and Pielou’s evenness (*E*) diversity indices were calculated using the following formula; *D* = 1-Σ (pi)^2^, *H* = -Σ*pi* log(*pi*), where *pi* = proportion of frequency of the ith phylotype in a sample. Phylotypes evenness was calculated as; *E* = *H*/log(*S*). Where *H* = Shannon Wiener diversity and *S* = phylotypes richness, i.e., total number of phylotypes. Fungal community diversities were compared and the specific levels of taxonomic resolution (rarefaction) were determined. The coverage saturation (*C*) was also calculated in order to verify the sufficiency of the sampling effort: *C* = 1 – (*n1*/*N*), where *n1* is the number of phylotypes that occurred once, and *N* is the total number of phylotypes examined. In order to determine similarity associations between fungal communities among the different samples, a dendrogram was constructed on the basis of a similarity matrix using Morisita–Horn’s similarity coefficient (Magurran, 2004). Analyses were conducted using the “vegan” package in R software (R Development Core Team, 2010).

## Results

### Molecular Characterization of Fungal Communities in Soil Samples

#### Fungal Community Composition and Phylogenetic Diversity

**Figure 1:**
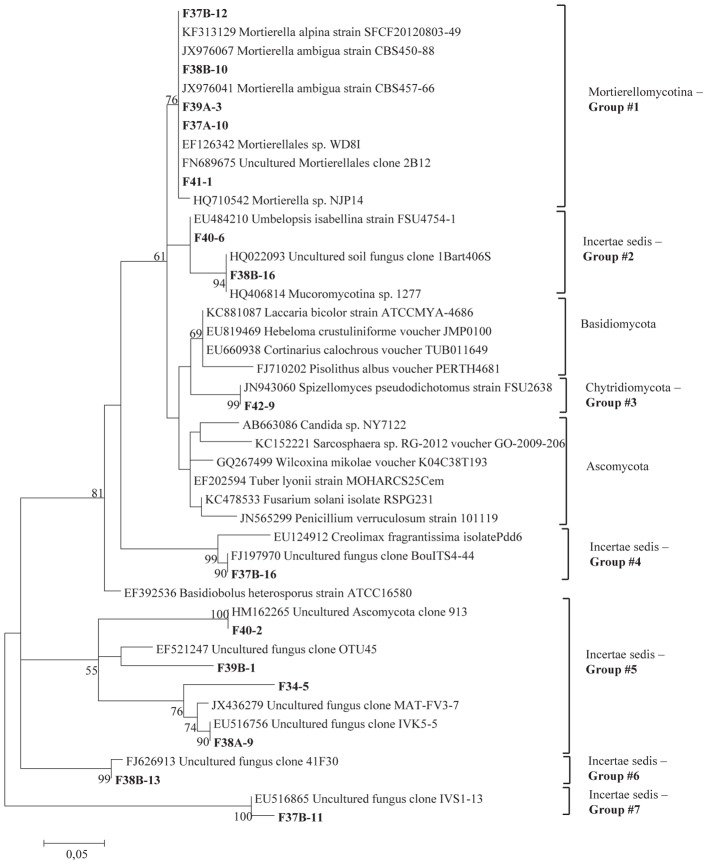
Phylogenetic tree showing maximum likelihood (ML) analysis of “*Uncertae sedis* fungi.” Bold sequences are from this study. Bootstrap percentage values (50%) generated from 1000 replicates from ML and posterior probabilities (>50%) from Bayesian analysis are shown as (ML bootstrap value/Bayesian posterior probabilities).

**Figure 2:**
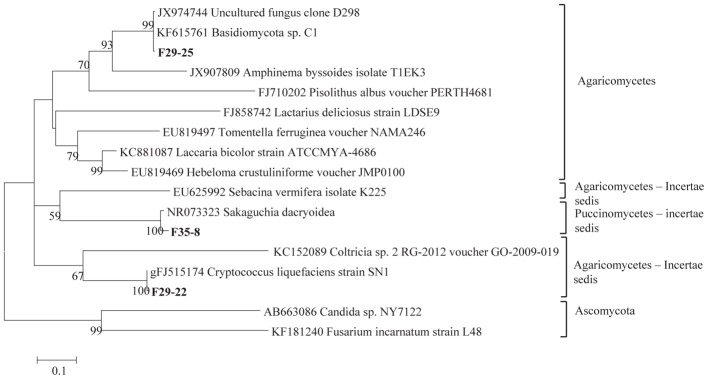
Phylogenetic tree showing ML analysis of Basidiomycota. Bold sequences are from this study. Bootstrap percentage values (50%) generated from 1000 replicates from ML and posterior probabilities (>50%) from Bayesian analysis are shown as (ML bootstrap value/Bayesian posterior probabilities).

**Figure 3:**
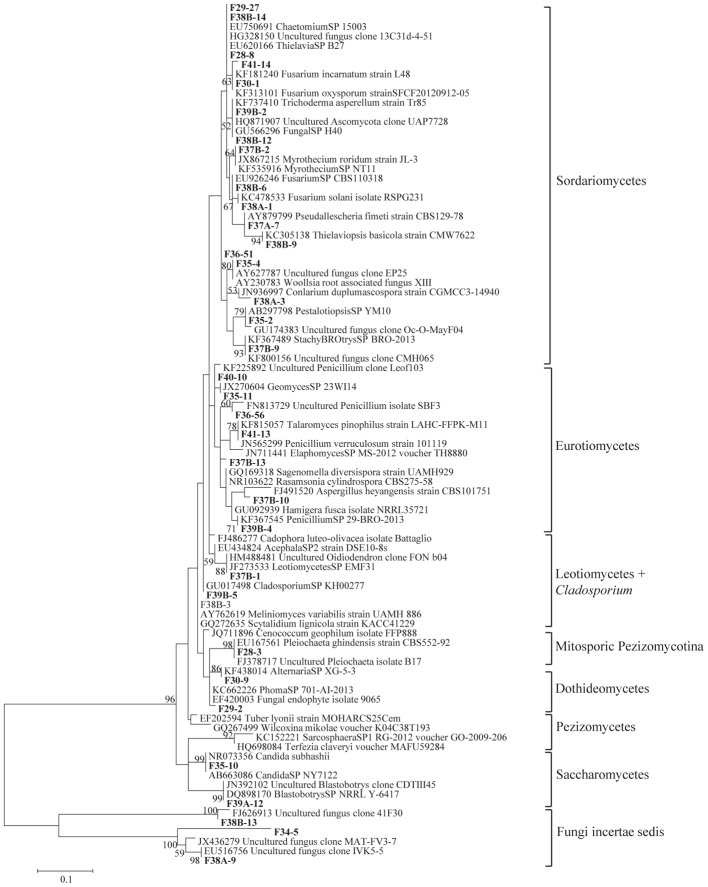
Phylogenetic tree showing ML analysis of Ascomycota. Bold sequence are from the study. Bootstrap percentage values (50%) generated from 1000 replicates from ML and posterior probabilities (>50%) from Bayesian analysis are shown as (ML bootstrap value/Bayesian posterior probabilities).

The total community DNA isolated from soil samples was of high molecular weight (700–800 bp by using the primer pair ITS1F-ITS4) and sufficient purity for successful amplification of ITS rDNA fragments. The ITS rDNA fragments were obtained from all DNA samples by direct PCR amplification. The majority of fungal ITS rDNA sequences (68.34%) had high sequence similarity, up to 100%, with those of environmental fungi or known species in the NCBI database. However, 5% of the sequences in our database could not match more than 500 bp with sequences in the NCBI database; most of these sequences matched members of Fungi incertiae sedis. Eighteen clone libraries were generated from bulk and rhizosphere soil samples and 8 to 17 clones were successfully sequenced per library. Phylogenetic assignment of phylotypes was performed according to best sequence matches based on BLASTn analyses. Data obtained from BLAST analyses are summarized in Table 1. We globally obtained 48 OTUs among the 217 classifiable clone sequences (Table 1). The Ascomycota, Basidiomycota, traditional Zygomycota and traditional Chytridiomycota represented the majority of fungal sequences derived from our clone libraries (Table 1). However, a large number of sequences that matched members of Fungi incertae sedis was found. Because of the taxonomic distances between these phyla, the phylogenetic trees were inferred separately (Figure 1–3).

**Table 1:** BLAST results in Genebank database for the representing clones sequences from soil fungal communities.

Sample label	Representative phylotype (clone)	Putative classification	Most closely related fungal sequence (accession number)	Isolation source	% identity
**Incertae sedis and Mortierellomycotina fungi**					
Ps-Ap-LT-1	F37B-12	Fungi incertae sedis; Mortierellomycotina; Group 1	*Mortierella ambigua* (JX976067)	Soil	99
Ps-Ap-LT-2	F38B-10	Fungi incertae sedis; Mortierellomycotina; Group 1	*Mortierella ambigua* (JX976041)	Soil	99
Ps-Ap-LT-3	F39A-3	Fungi incertae sedis; Mortierellomycotina; Group 1	*Mortierella sp*. (HQ710542)	Soil	98
Ps-Ap-SA-2	F41-1	Fungi incertae sedis; Mortierellomycotina; Group 1	*Mortierella alpina* (KF313129)	Pine root	99
Ps-Ap-LT-1	F37A-10	Fungi incertae sedis; Mortierellomycotina; Group 1	*Mortierellales sp*. (EF126342)	Soil	99
Ps-Ap-SA-1	F40-6	Fungi incertae sedis; Mucoromycotina; Group 2	*Umbelopsis isabellina* (EU484210)	–	99
Ps-Ap-LT-2	F38B-16	Fungi incertae sedis; Group 3	Uncultured soil fungus (HQ022093)	Soil	89
Ps-Ap-SA-3	F42-9	Chytridiomycota incertae sedis; Group 4	*Spizellomyces pseudodichotomus* (JN943060)	–	83
Ps-Ap-LT-1	F37B-16	Fungi incertae sedis; Group 5	Uncultured fungus (FJ197970)	Soil	88
Ps-Ap-LT-3	F39B-1	Fungi incertae sedis; Group 6	Uncultured fungus (EF521247)	Spruce forest	90
Ps-Ap-SA-1	F40-2	Fungi incertae sedis; Group 6	Uncultured Ascomycota (HM162265)	Grass roots	92
Ps-Ap-LT-2	F38A-9	Fungi incertae sedis; Group 6	Uncultured fungus (EU516756)	Soil	92
Ps-Bp-1	F34-5	Fungi incertae sedis; Group 6	Uncultured fungus (JX436279)	Microbial mat	91
Ps-Ap-LT-1	F37B-11	Fungi incertae sedis; Group 7	Uncultured fungus (EU516865)	Soil	97
Ps-Ap-LT-2	F38B-13	Fungi incertae sedis; Group 8	Uncultured fungus (FJ626913)	Rhizosphere	83
**Basidiomycota fungi**					
LT-S-2	F29-22	Agaricomycotina; Tremellomycetes	*Cryptococcus liquefaciens* (FJ515174)	–	99
Ps-Bp-2	F35-8	Pucciniomycotina; Cystobasidiomycetes	*Sakaguchia dacryoidea* (NR073323)	–	95
LT-S-2	F29-25	Basidiomycota incertae sedis	*Basidiomycota sp*. (KF615761)	Host = “citrus”	99
**Ascomycota fungi**					
LT-S-3	F30-1	Sordariomycetes; Hypocreales; Fusarium Clade 1	*Fusarium incarnatum* (KF181240)	Host = “alfalfa”	99
Ps-Ap-SA-2	F41-14	Sordariomycetes; Hypocreales; Fusarium Clade 1	*Fusarium oxysporum* (KF313101)	Pine root	99
Ps-Ap-LT-2	F38B-6	Sordariomycetes; Hypocreales; Fusarium Clade 2	*Fusarium sp*. (EU926246)	–	99
Ps-Ap-LT-2	F38A-1	Sordariomycetes; Hypocreales; Fusarium Clade 2	*Fusarium solani* (KC478533)	Soil	99
Ps-Ap-LT-3	F39B-2	Sordariomycetes; Hypocreales	*Trichoderma asperellum* (KF737410)	–	100
**Ascomycota fungi**					
Ps-Ap-LT-1	F37B-2	Sordariomycetes; Hypocreales	*Myrothecium sp*. (KF535916)	Soil	100
LT-S-2	F29-27	Sordariomycetes; Sordariales	*Chaetomium sp*. (EU750691)	–	98
LT-S-1	F28-8	Sordariomycetes; Sordariales	*Thielavia sp*. (EU620166)	Biosolids compost	99
Ps-Ap-LT-2	F38B-12	Sordariomycetidae; incertae sedis	Fungal sp. (GU566296)	Soil	99
Ps-Ap-LT-2	F38B-14	Sordariomycetidae; incertae sedis	*Myrothecium roridum* (JX867215)	Stems of *Tribulus terrestris*	90
Ps-Ap-LT-1	F37B-9	Sordariomycetes; Hypocreales	*Stachybotrys sp*. (KF367489)	Water	95
Ps-Bp-2	F35-2	Sordariomycetidae; Xylariales	*Pestalotiopsis sp*. (AB297798)	Wood tissue	89
Ps-Ap-LT-1	F37A-7	Sordariomycetes; Microascales	*Pseudallescheria fimeti* (AY879799)	–	97
Ps-Ap-LT-2	F38B-9	Sordariomycetes; Microascales	*Thielaviopsis basicola* (KC305138)	–	100
Ps-Ap-LT-2	F38A-3	Sordariomycetidae; incertae sedis	*Conlarium duplumascospora* (JN936997)	Submerged wood	93
Ps-Bp-2	F35-4	Sordariomycetidae; incertae sedis	Woollsia root associated fungus (AY230783)	*Woollsia pungens*	89
Ps-Ap-SA-1	F40-10	Eurotiomycetes; Eurotiales	Uncultured Penicillium (KF225892)	Soil	88
**Ascomycota fungi**					
Ps-Ap-LT-1	F37B-13	Eurotiomycetes; Eurotiales	*Rasamsonia cylindrospora* (NR_103622)	–	84
Ps-Ap-SA-2	F41-13	Eurotiomycetes; Eurotiales	*Talaromyces pinophilus* (KF815057)	Rhizoplane	99
Ps-Ap-LT-3	F39B-4	Eurotiomycetes; Eurotiales	*Penicillium sp*. (KF367545)	Water	99
Ps-Ap-LT-1	F37B-10	Eurotiomycetes; Eurotiales	*Hamigera fusca* (GU092939)	–	84
Ps-Bp-2	F35-11	Eurotiomycetes; Onygenales	*Geomyces sp*. (JX270604)	Soil from bat hibernaculum	99
Ps-Ap-LT-2	F38B-3	Leotiomycetes; mitosporic Leotiomycetes	*Scytalidium lignicola* (GQ272635)	–	96
Ps-Ap-LT-1	F37B-1	Leotiomycetes; unclassified Leotiomycetes	*Leotiomycetes sp*. (JF273533)	Evergreen broad-leaved forest	92
Ps-Ap-LT-3	F39A-12	Saccharomycotina; Saccharomycetes	*Blastobotrys sp*. (DQ898170)	–	96
Ps-Bp-2	F35-10	Saccharomycotina; Saccharomycetes	*Candida subhashii* (NR_073356)	–	99
Ps-Ap-LT-3	F39B-5	Dothideomycetes; Capnodiales	*Cladosporium sp*. (GU017498)	–	99
LT-S-2	F29-2	Dothideomycetes; Pleosporales	*Phoma sp*. (KC662226)	Host = “Soybean”	99
LT-S-3	F30-9	Dothideomycetes; Pleosporales	*Alternaria sp*. (KF438014)	Green house	100
LT-S-1	F28-3	Mitosporic Pezizomycotina	Pleiochaetaghindensis (EU167561)	–	97

*The codes of the different soil samples are as follow: LT-S, Lonely tree composite rhizospheric soil; Ps-Bp, Planting Soil before planting *Vachellia pachyceras* seedlings (non-rhizospheric crude commercial soil mix); Ps-Ap-LT, Planting Soil after planting Lonely Tree seedlings (rhizospheric commercial soil mix of local *V. pachyceras*); Ps-Ap-SA, Planting Soil after planting Saudi Arabia *Vachellia pachyceras* seedlings (rhizospheric commercial soil mix of non-local *V. pachyceras*). The numbers following the codes indicate the different replicates*.

Sequence analysis showed that members of the phylum Ascomycota were the most common group in this study (Table 1). Twenty-three fungal sequences belonged to this group, which showed high similarities to their closest relatives. Of the Ascomycota (Figure 3), Sordariomycetes were by far the most abundant (16 OTUs), followed by the Eurotiomycetes (6 OTUs), Leotiomycetes (2 OTUs), Saccharomycetes (2 OTUs), Dothideomycetes (3 OTUs) and mitosporic Pezizomycota (1 OTU). Mortierellomycota were the second largest contributor in terms of phylogenetic diversity with 5 OTUs belonging to this sub-phylum (Figure 1). Traditional Chytridiomycota were detected but were rare in this study. Besides those fungal phyla, Basidiomycota were represented by 3 OTUs belonging to the Agaricomycetes (Figure 2). The remaining sequences, mostly affiliated to uncultured fungi from diverse environments, matched members of Fungi incertae sedis (Table 1 and Figure 1). They were divided into six groups outlined in Figure 1 and were represented by 10 OTUs. It is also remarkable to note that Sordariomycetes were widely distributed across the different soil samples, whereas other classes such as Saccharomycetes were almost exclusively found in bulk soils. The most abundant genera and species were *Mortierella* sp. (Mortierellomycotina) and *Fusarium* sp. (Sordariomycetes).

#### Rhizosphere Effect

The degree to which fungal communities are influenced in the rhizospheres of plants compared to the non-rhizospheric control bulk soils was analyzed at a level of class. A differential distribution pattern of the detected fungal taxa among the different soil rhizospheres was observed at both field and nursery environments (Table 2). In more detail, the rhizosphere soils (LT-S) were clearly dominated by fungal phylotypes of the mitosporic Pezizomycotina class (72.5% of the communities) followed by members of the Dothideomycetes, which accounted for 17.02%. The remaining individual classes were relatively rare and accounted for up to 6.3% of the fungal communities. In contrast, the non-rhizosphere (bulk soil) samples (LT-CS) showed the lowest biodiversity in terms of community structure (Table 1). They were represented by fungal phylotypes of a single class; Basidiomycetes, which had clearly increased its abundance in bulk soil samples (100% of fungal communities).

**Table 2:** Frequency and diversity indices for the mean of samples from different origins.

Taxonomic groups	LT-S	LTCS	Ps-Bp	Ps-Ap-LT-A	Ps-Ap-LT-B	Ps-Ap-SA
***Incertae sedis and Mortierellomycotina tree***					
Group 1 (Mortierellomycotina)	0	0	0	14	6	13
Group 2	0	0	0	0	0	1
Group 3	0	0	0	0	1	0
Group 4	0	0	0	0	0	1
Group 5	0	0	0	0	1	0
Group 6	0	0	1	1	1	0
Group 7	0	0	0	0	1	0
Group 8	0	0	0	0	1	0
***Basidiomycota tree***					
Basidiomycota (Agaricomycetes)	2	30	1	0	0	0
***Ascomycota tree***					
Sordariomycetes	3	0	21	5	11	2
Eurotiomycetes	0	0	2	6	3	10
Leotiomycetes	0	0	0	0	4	0
Saccharomycetes	0	0	14	1	0	0
Dothideomycetes	8	0	0	0	1	0
Mitosporic Pezizomycotina	34	0	0	0	0	0
Total abundance	47	30	39	27	30	27
OTU richness (S)	8	1	6	11	22	10
Shannon–Wiener index (S)	1,009	0,000	1,337	2,074	3,014	1,742
Simpson index (1-D)	0,452	0,000	0,682	0,837	0,947	0,738
Evenness Pielou (E)	0,485	NaN	0,746	0,865	0,975	0,757
Rarefaction (20 individuals)	4,539	1,000	4,775	9,323	16,350	8,125
Coverage (C)	1,000	1,000	0,945	0,926	0,800	0,926

*The codes of the different soil samples are: LT-S, Lonely tree composite rhizospheric soil; LT-CS, Lonely tree non-rhizospheric control soil; Ps-Bp, Planting Soil before planting *V. pachyceras* seedlings (non-rhizospheric crude commercial soil mix); Ps-Ap-LTA and Ps-Ap-LTB, Planting Soil after planting Lonely Tree seedlings (rhizospheric commercial soil mix of local *V. pachyceras*); Ps-Ap-SA, Planting Soil after planting Saudi Arabia *V. pachyceras* seedlings (rhizospheric commercial soil mix of non-local *V. pachyceras*)*.

The Ps-Ap-LT soils (grown in optimal nursery conditions) contain fungal phylotypes of at least four dominant classes: Sordariomycetes (36.7%), Leotiomycetes (13.3%), and Eurotiomycetes (10%) for the sample B and, Sordariomycetes (18.5%) and Eurotiomycetes (22.2%) for the sample A. Close relatives of Mortierellomycotina incertae sedis fungi were also common in these soil samples with 20 and 51.85% for the Ps-Ap-LT sample B and sample A, respectively. The other classes are of minor importance and accounted for up to 3.4% of the soil fungal communities. The Ps-Ap-SA treatment was dominated by fungal phylotypes of the Mortierellomycotina incertae sedis (48.15% of fungal communities), followed by members of the Eurotiomycetes (37.04%). The remaining classes were relatively rare and accounted for up to 7.4% of the soil communities. The non-rhizosphere (bulk soil) samples (Ps-Bp) were affiliated to three main classes of Ascomycota: Sordariomycetes (54%), Saccharomycetes (36%) and Eurotiomycetes (5.1%). Other phylotypes from this clone library are of minor importance, with sequences having nearest hits to members of Agaricomycetes (2.5% of fungal communities) and incertae sedis Group 6 (2.5%).

Globally taken, the rhizosphere samples LT-S, Ps-Ap-LT-A, Ps-Ap-LT-B, and Ps-Ap-SA treatments, with 8, 11, 22, and 10 OTUs, respectively, showed the highest richness of fungal communities. The field and nursery bulk soil samples disclosed 1 and 6 OTUs, respectively. Indices of Shannon (H) for diversity and Simpson (1–D) for evenness were also calculated, and data were broadly in agreement with those reported with the richness index (Table 2). Collectively, data from this study illustrate that Bulk soil samples were consistently less even than rhizosphere soils. Difference in coverage were also marked among the different samples, the nursery rhizosphere soils (Ps-Ap-LT and Ps-Ap-SA) showing lesser values of rarefaction index, suggesting that sample efforts are further needed to saturate the organismal richness in these samples.

**Figure 4:**
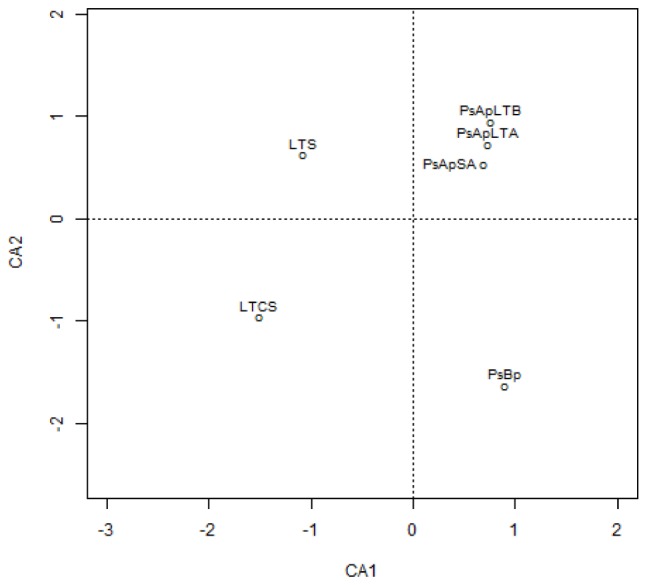
Correspondence analysis of the different fungal communities in soil rhizosphere of *Vachellia pachyceras* in diverse conditions. Soil samples are positioned along the first two DA axes, where Eigen values are 0.9919 for CA1 and 0.9536 for CA2. The codes of the different soil samples are: LT-S, Lonely tree composite rhizospheric soil; LT-CS, Lonely tree non-rhizospheric control soil; Ps-Bp, Planting Soil before planting *V. pachyceras* seedlings (non-rhizospheric crude commercial soil mix); Ps-Ap-LTA and Ps-Ap-LTB, Planting Soil after planting Lonely Tree seedlings (rhizospheric commercial soil mix of local *V. pachyceras*); Ps-Ap-SA, Planting Soil after planting Saudi Arabia *V. pachyceras* seedlings (rhizospheric commercial soil mix of non-local *V. pachyceras*).

**Figure 5:**
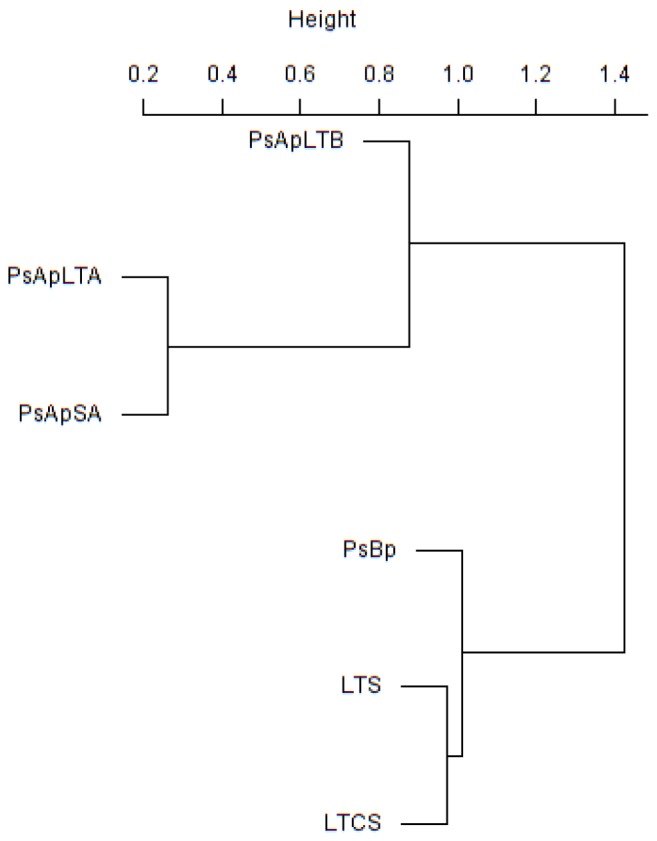
Cluster analysis based on Morisita-Horn similarity coefficient for different fungal communities in soil rhizosphere of *V. pachyceras* in diverse conditions. The codes of the different soil samples are: LT-S, Lonely tree composite rhizospheric soil; LT-CS, Lonely tree non-rhizospheric control soil; Ps-Bp, Planting Soil before planting *V. pachyceras* seedlings (non-rhizospheric crude commercial soil mix); Ps-Ap-LTA and Ps-Ap-LTB, Planting Soil after planting Lonely Tree seedlings (rhizospheric commercial soil mix of local *V. pachyceras*); Ps-Ap-SA, Planting Soil after planting Saudi Arabia *V. pachyceras* seedlings (rhizospheric commercial soil mix of non-local *V. pachyceras*).

In addition, principal component (PCA) and Cluster analyses (based on the Morisita–Horn’s similarity coefficient) were conducted to compare similarities between fungal communities of the different samples. According to these analyses, marked differences exist in the fungal community compositions of soil samples: two main clusters were distinguished (Figure 5). The first cluster included the rhizosphere soils collected from both *V. pachyceras* (Ps-Ap-LT-A, Ps-Ap-LT-B, and Ps-Ap-SA treatments) grown in nursery conditions and, the second cluster comprised the Lonely Tree (LT-S) and bulk soil (LT-CS and Ps-Bp) samples. However, Figure 4 showed that nursery bulk soils (Ps-Bp) were segregated from LT-S and LT-CS samples, thus suggesting that their soil fungal communities were different. Ps-Ap-LT was replicated in order to verify that the sample effort is reliable. Figure 5 clearly shows that both replicates were grouped together and roughly included in the same branch cluster.

### Molecular Characterization of Arbuscular Mycorrhizal Fungal Communities From Root System

DNA extractions were performed using the Plant DNeasy kits (Qiagen, ON) from roots stored in CTAB. The expected 750–800 bp PCR fragment was obtained with the nested PCR with LR1-FLR 4. From the total DNA amplicons, 15 clone libraries were produced, and 3 to 14 clones were successfully sequenced per library (Table 3). Among all 133 clones sequenced, 10 phylotypes could be identified (Table 3).

**Table 3:** BLAST results for the representing clone’s sequences from AMF communities in roots and statistical analysis.

Phylotypes	Clones	Accession	Identity	% Similarity	LT-AM	N-LT-AM	N-SA-AM
no.	representative						
1	13A-4	JX999965	Glomeromycota F84 clone B	98	21	18	10
2	6B-12	JX999971	Glomeromycota F80 clone F	99	13	12	2
3	15C-11	HE858411	Uncultured glomus clone FW3-5	95	2	1	1
4	22B-6	HE817882	Rhizophagus irregularis DAOM197198 clone pHS052-37	98	8	0	9
5	22B-1	FM992381	Glomus sp. Att690-23 DAOM:197198	98	0	0	1
6	14C-3	JF439202	Glomus sp. 7 SUN-2011 isolate 07_10_1	94	0	4	0
7	22B-11	AM040435	Glomus sp. Rp2 clone 2	98	0	4	21
8	13A-1	KC411228	Uncultured glomerales clone B08_06	92	0	2	0
9	13A-6	KF849658	Uncultured glomus clone AM178	99	0	3	0
10	R1-25	JN937539	Glomeromycota sp. OTU3 DJMC-2012	95	0	0	0
11	5B-13	JF439189	Glomus sp. 7 SUN-2011 isolate 08_40_1	95	1	0	0
			Nb of libraries		5	5	5
			Shannon–Wiener diversity index		1,244	1,566	1,327
			Species richness (S)		5	7	6
			Total abundance		45	44	44
			Simpson diversity index	D:	0,335	0,265	0,324
				1-D:	0,665	0,735	0,676
				1/D:	2,982	3,767	3,083
			Evenness (Pielou)		0,773	0,805	0,741

*The codes of the different soil samples are: LT-AM, Lonely Tree Arbuscular Mycorrhizal root; N-LT-AM, Nursery grown Lonely Tree Arbuscular Mycorrhizal root (local *V. pachyceras*); N-SA-AM, Nursery grown Saudi Arabia Arbuscular Mycorrhizal root (non-local *V. pachyceras*)*.

**Figure 6:**
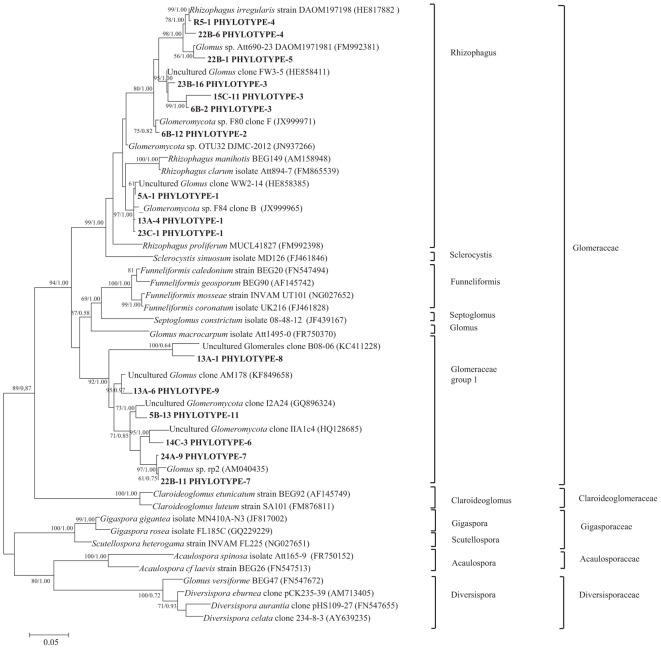
Phylogenetic tree showing ML analysis of arbuscular mycorrhizal fungi. Bootstrap percentage values (50%) generated from 1000 replicates from ML and posterior probabilities (>50%) from Bayesian analysis are shown as (ML bootstrap value/Bayesian posterior probabilities). Bold sequences are from this study.

**Figure 7:**
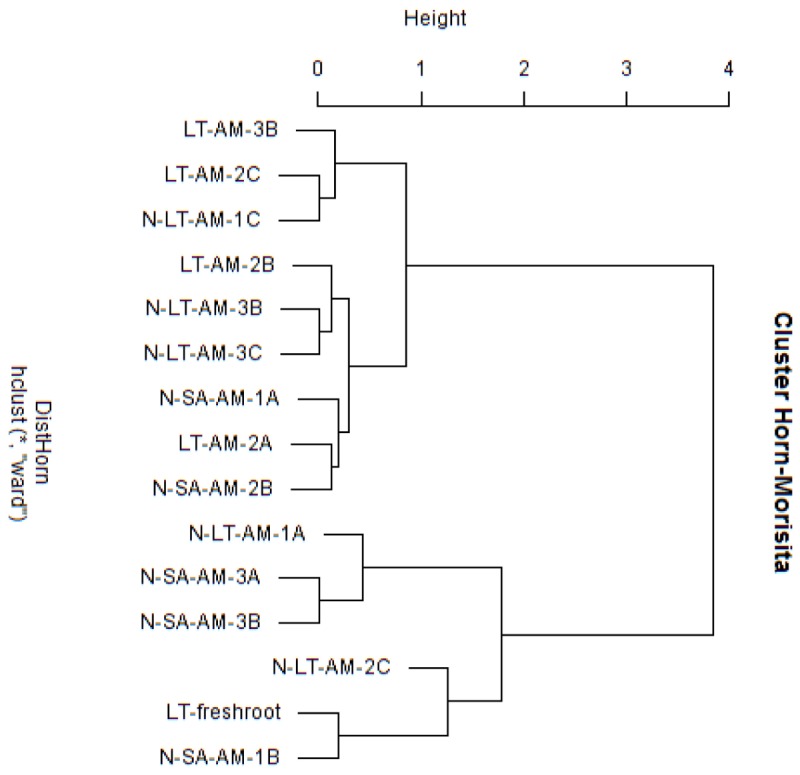
Cluster analysis based on Morisita-Horn similarity coefficient for different arbuscular mycorrhizal fungal communities in roots of *V. pachyceras* in diverse conditions. The codes of the different soil samples are: LT-AM, Lonely Tree Arbuscular Mycorrhizal root; N-LT-AM, Nursery grown Lonely Tree Arbuscular Mycorrhizal root (local *V. pachyceras*); N-SA-AM, Nursery grown Saudi Arabia Arbuscular Mycorrhizal root (non-local *V. pachyceras*). LT, Lonely Tree; The clone number were added at the end of the sample codes.

Nucleotide BLAST results in GenBank database for the representing clones sequenced are shown in Table 3. First, analysis was performed excluding “Uncultured/environmental sample sequences.” If similarity percentage between the query and GenBank sequence was below 95%, nucleotide BLAST was computed again including “Uncultured/environmental sample sequences.” This was the case for phylotypes #3, 8 and 9, for which similarity with NCBI-deposited sequences was below 95%. Thus, the matching “Uncultured/environmental sample sequences” was included in further analyses. Branches that delimit phylotypes and their matching sequence were well supported, with 73 to 99% of bootstraps values and 0.67 to 1.00 of Bayesian posterior probabilities. Tree topology acquired after computing NJ and ML analyses were similar. Figure 6 present the ML tree on which is observed a different branching pattern between ML and Bayesian analyses at the family level, so it does not affect interpretation. Phylogenetic tree (Figure 6) showing molecular phylogenetic analysis obtained by maximum likelihood (ML) analysis of arbuscular mycorrhizal fungi. The most abundant and diversified main group was found within the family Glomeraceae. However, in the phylogenetic tree, on the right side it is showing two taxonomic groups. In the first group, it is assigned into two sub-groups clustering the genera *Rhizophagus* and *Sclerocystis* in which 5 different phylotypes included with the exception of phylotype 4, where a sequence of *Rhizophagus irregularis* DAOM 197198 was included in the branch group, no other type was included in the phylotype branches. Thus, a species could not be assigned to these groups. In the second group, only a single sub-group assigned and clustering as Glomeraceae group-1, in which another 5 phylotypes with unclassified taxonomic group included. According to cluster analysis in Figure 7, a closer relationships exist among AMF communities from Lonely Tree (LT-AM) and (N-LT-AM) than the Saudi Arabia tree (N-SA-AM). For AMF composition and frequency in the different communities (Table 3), it can be observed that the most common phylotype for all root types is the phylotype no. 1. Phylotype no. 2 is very abundant for LT-AM and N-LT-AM, and phylotype no. 7 is abundant for N-SA-AM. The highest diversity indices are found in seedlings N-LT-AM, followed by N-SA-AM and LT-AM.

## Discussion

### Fungal Community Structures

To our knowledge, this study is the first attempt using PCR based molecular approach to reveal the diversity of general fungal communities and AM fungi associated with the only surviving nationally important tree specises – *V. pachyceras* in Kuwait desert. The present work provides us with a preview of general fungal communities associated with rhizosphere and non-rhizosphere soils of *V. pachyceras* from the Sabah Al-Ahmad Natural Reserve of the Kuwait desert and nursery growing media. Data showed that despite a hostile climate that the Kuwait desert represents, the studied area maintained surprisingly diverse fungal biodiversity, largely consisting of fungi adapted to harsh environmental conditions exists in desert. Our results are in agreement with a recent report showing unpredictably huge fungal biodiversity in the Middle Eastern desert soils (Murgia et al., 2018). For the overall community composition of this study site, the majority of fungal sequences recovered were classified into four major fungal phyla: Ascomycota, Basidiomycota, Zygomycota and Chytridiomycota, which account for 80% of the OTUs. Consistent with the previous studies (El-Said and Saleem, 2008; Bates et al., 2012; Sterflinger et al., 2012; Abed et al., 2013; Bastida et al., 2013), Ascomycota was the most abundant phylum (48%), whereas Basidiomycota accounted for a much smaller percentage of the community (10.4%). A large body of investigation has supported this dominance of Ascomycota fungi in arid and semi-arid soils. In a recent study, soil sample from Saudi Arabia and Jordan deserts revealed that the most abundant fungal phyla was Ascomycota similar to our results (Murgia et al., 2018). Abed et al. (2013) showed that in the arid desert of the Arabian Peninsula Ascomycota represents >86% of their pyrosequencing reads, while forming more than 98% of the observed isolates. Using molecular techniques, Green et al. (2008) and Bates and Garcia-Pichel (2009) reported dominance of Ascomycota fungi in soils from the Chihuahuan desert (83.3% of 989 sequences) and in Colorado Plateau (87–91% of 135 sequences), respectively. Other data from Grishkan et al. (2006) have also shown this dominance in the Negev desert (98% of 58 species). The results, however, are disclosed to those of a few scale surveys of desert soils (Connell et al., 2006; Fell et al., 2006) where Basidiomycota was the dominant phylum. These analyses and our data from the Kuwait desert indicate the variations of fungal community composition among desert lands. Molecular analyses data of this study clearly demonstrated these variations in soil fungal community composition and reflected in the non-rhizospheric control LT bulk soil, which had very low levels of fungal composition with OTUs Richness – 1 and Shannon–Weiner Index (S) – 0.0. In all other soils, such as nursery soils tested were found much higher OTUs Richness and higher Shannon–Weiner Index as these soils have relatively higher organic matter and maintained in optimal growing conditions or have vegetation effects. Chytridiomycota and Zygomycota fungi seem to be underrepresented, compared to the number of sequences in the Ascomycota. Similar results have been previously reported in Omani desert and Chihuahuan desert by Green et al. (2008).

Of the Ascomycota, the most OTU-rich fungal classes were Sordariomycetes and Eurotiomycetes. The former is the only class showing high diversity in each soil sample, indicating their high ecological plasticity. Described members of the Sordariomycetes are assumed to be cosmopolitan, and function as plant and animal pathogens, endophytes of plants, and saprobes involved in decomposition and nutrient cycling (Brunner et al., 2011; Abed et al., 2013; Qin et al., 2014). In the present study, the majority of OTUs sequences belonging to the Sordariomycetes matched previously described species and was related to the following genera: *Fusarium*, *Myrothecium*, *Trichoderma*, *Chaetomium*, *Thielavia*, *Stachybotrys*, *Pestalotiopsis*, *Pseudallescheria*, *Thielaviopsis*, *and Conlarium*. They mostly belonged to the orders Hypocreales, Sordariales, Xylariales, and Microascales. These fungi most likely play a role in organic plant material breakdown in a symbiotic or mutualistic relationship with plant species (Meiser et al., 2014; Powell et al., 2015).

The largest assemblages of fungal OTUs belonging to the Eurotiomycetes comprise members of the genera *Rasamsonia*, *Talaromyces*, *Penicillium*, *Hamigera*, *and Geomyces*. They belonged to the orders Euritiales and Onygenales, which include cellulolytic soil saprophytes fungi (Sugiyama et al., 1999; Berbee, 2001). Most of the OTUs in the Leotiomycetes class belonged to the genera *Scytalidium* and *Leotiomycetes* whereas most of the OTUs in the Saccharomycetes class belonged to the genera *Blastobotrys* and *Candida*. Saccharomycetes class includes genera of ascomycetous yeasts and one pathogenic on human (*Candida*) (Berbee, 2001).

Dothideomycetes OTUs were assembled into three genera *Cladosporium*, *Phoma*, and *Alternaria*. They mostly belonged to the order Pleosporales. Fungi belonging to this order (most notably *Alternaria*) were used before as indicative of desert settings (Bates et al., 2012; Sterflinger et al., 2012). This is mainly because they typically have darkly pigmented spores or hyphae stained with allomelanins, which may provide protection from excessive exposure to UV radiation (Bates et al., 2012). They can often be found as endophytes or epiphytes of living plants, and also as saprobes degrading cellulose, keratin and other complex carbohydrates in dead or partially digested plant matter in leaf litter (Porras-Alfaro et al., 2010; Nguyen et al., 2011; Abed et al., 2013). Nevertheless, species of the genus *Aspergillus*, being very abundant in Israel (Grishkan and Nevo, 2010), Saudi Arabia and Libya (Abdel-Hafez, 1981, 1982), were missing in our samples.

Basidiomycota have been reported to be diverse in soil ecosystems (Buée et al., 2009), but is not confirmed here. We showed that only 6.25% of the OTUs belong to this phylum. Most sequences matched species belonging to the *Cryptococcus* and *Sakaguchia* genera. These genera are known to comprise a number of human associated species, as either opportunists or pathogens (de Hoog et al., 2000). Dominance of yeast genera including *Cryptococcus* genus is also reported in Antarctica in a study by Arenz and Blachette (2011).

Most OTUs sequences in the Zygomycota and Chytridiomycota phyla matched species belonging to the *Mortierella* and *Spizellomyces* genera, reportedly common fungal groups in soils (Brown and Jumpponen, 2014; Zhang et al., 2014). Members of the former are reported to mineralize readily available dissolved organic substrates rather than breaking down soil litter polymers (Schmidt et al., 2008; Brown and Jumpponen, 2014), while the later has been found to infect spores of arbuscular mycorrhizal fungi (Ross and Ruttencutter, 1977; Daniels, 1981). OTU sequences belonging to the chytrids were also detected in soil crusts of the Sultanate of Oman in the Arabian Desert (Abed et al., 2013). However, their detection in arid deserts, although in low abundance (<3% of total OTUs) is intriguing and more research is needed in order to determine the ecological role of aquatic Chytridiomycetes fungi associated with desert lands.

Several OTUs sequences mostly affiliated to uncultured fungi from diverse environments, matched members of Fungi incertae sedis. These sequences were assembled into six groups and seem to correspond to a well-supported clad of Ascomycota, equivalent to endophytic of dark septate fungi. The detection of dark-colored fungi is a typical feature of desert soils mainly because of their ability to survive high solar radiation and temperature (Grishkan et al., 2006). Further analyses are required to explore functional attributes of these fungal species and to classify them at the genus level.

### Rhizosphere Effect on Soil Fungal Communities

Microbial activity in deserts are concentrated in brief periods of high soil wetness following rainfall events and are expected to be greater in rhizosphere soils, depending on the plant type (Hollister et al., 2010; Zhang et al., 2014). We sought to investigate the rhizosphere effect of the LT *V. pachyceras* and the non-local Saudi Arabia *V. pachyceras*, on soil fungal community structures by assessing fungal diversity in soils from the same species grown in optimal nursery environments. The foremost aspect of fungal community structures that is so clear as to be unassailable in this study is the rhizosphere effect, with more fungal diversity and OTUs richness associated with both field and nursery rhizosphere soils. In contrast, a less diverse fungal community was found in the bulk soils. Such plant-dependent enrichment has received increasing support recently (Xu et al., 2012; Meiser et al., 2014; Welc et al., 2014; Zhang et al., 2014). Indeed, plant may exude a variety of carbon sources that can be consumed by fungal communities, thus creating more niches for them to occupy and promoting increased fungal richness (Weber et al., 2011). However, ideal nursery growing conditions with nutrient availability may also favor microbial population in soils. In the absence of root plants, the nutrient limitations and the combined disturbance of climatic conditions in deserts could preclude the growth and hamper the hyphal proliferation of many fungi (Meiser et al., 2014). Thus, only fungal species that might be highly specialized to such an ecological niche could be found (Bates et al., 2012). Those data and the results from fungal community structure, pointed out the complexity of the interdependency of soil microbial diversity with plant species.

Although the nursery experiment was not designed to specifically test the impacts of soil properties on the soil fungal communities, the results of this study suggest that they have an effect. Compared to the field soils, fungal communities in the nursery soils were more diverse owing to soil chemical richness and substrate. This is analogous to an edaphic condition effect and has been reported in other studies (de Castro et al., 2008; Qin et al., 2014; Welc et al., 2014). Thus, we hypothesized that by providing a nutritional resource and stable substrate, nursery sustains the growth of many more fungi than do field environments. Nevertheless, from this study, this edaphic condition effect seems to be overwhelmed by the response of fungi to the root plant presence. It is not surprising that differences in soil chemical properties may drive the observed higher fungal communities and diversity associated with nursery potting soils and LT rhizospheric soils compared to LT non-rhizospheric control soils.

### Arbuscular Mycorrhizal (AM) Communities From Root System

This study is the first to use of molecular technique to reveal the AM fungal communities from the roots of *V. pachyceras* the only surviving native tree species in Kuwait desert and the roots of nursery grown *V. pachyceras* seedlings. Our attempt was to uncovering AM fungal community composition and obtained a baseline data that was previously unknown with this unique tree in Kuwait. Initial staining of roots confirmed the presence of AM fungi in the LT (*V. pachyceras*) and in the roots of nursery-grown *V. pachyceras* seedlings (Supplementary Material). This observation is further supported by the molecular characterization of AM communities associated with root systems of all the test plants of this study. Results from molecular characterization, a total of 10 phylotypes were identified, in which 5 phylotypes revealed with most abundant group from the family Glomeraceae with the presence of genera *Rhizophagus* formerly known as *Glomus* sp., suggesting specific recruitment preference of AM fungi partners by the single plants (Xu et al., 2017). However, another 5 phylotypes were also revealed with unclassified taxonomic group under Glomeraceae group 1. Furthermore, our result revealed that the diversity of AM fungi was more pronounced in the nursery-grown *Vachellia* seedlings than in the desert habitat of the LT roots. The identified phylotypes belonged to the Family Glomeraceae supporting the results of the examination of the spores in the soil, which morphologically resembles Glomeraceae (Supplementary Material). Nevertheless, the unclassified taxonomic group with five phylotypes revealed in this investigation suggests that further detailed analysis is required to elucidating the unclassified group fully. Similar to our findings, many studies have shown that AM fungi belonging to the genus *Glomus* and *Rhizophagus* were the most dominant in desert as well as other ecosystems, and most common AM fungi revealed throughout the world (Pande and Tarafdar, 2004; Shi et al., 2007; Al-Yahya’ei et al., 2011; Xu et al., 2017). The abundance of Glomeraceae in complex arid regions may be due to its ability to withstand harsh arid climatic conditions and resistance to high temperatures (Bever et al., 2009; Barto et al., 2011).

Even though AM fungal infection was detected morphologically under microscope from dried and thick roots typical for desert plants, DNA extraction was successful but the amplification and sequencing of such DNA samples was inadequate. Further standardization of amplification and sequencing is required for the desert AM fungal communities unique to Kuwait desert environment. Furthermore, it might be because of insufficient representation of AM fungal taxa in existing reference databases used. Nevertheless, the present research revealed a considerable diversity among the different root samples tested that has not been earlier depicted. The impact of environmental attributes on AM fungal communities that may influence shape these communities are still not adequately known (Xu et al., 2017). Data of this study showed that despite the harsh climate that prevails in the Kuwait desert, studied roots displayed the presence of AM fungal biota. However, the greatest diversity was noted in the roots of seedling from nursery, suggesting that diversity in the harsh desert conditions is lower compared to when seedlings were grown in nursery soil mix under ideal nursery conditions. It should be noted that not all sequences produced a match with a described well-known species; only the composition of the communities at the class level for the taxonomic rank could be characterized. However, it seems that soil factors may influence fungal composition and diversity more than tree species characteristics and their root system (Oehl et al., 2017). Although AM fungal spore counts are often low in arid soils and zero counts are common (Cui and Nobel, 1992; Requena et al., 1996; Titus et al., 2002). Data from this work demonstrate that a good number of AM fungal species exist in the Kuwaiti desert that need to be fully explored. However, this study established foundation work of the AM fungal communities associated with roots of *V. pachyceras* in Kuwait desert as well as nursery seedlings. This research delivers essential insight about ecological characteristics of *V. pachyceras*. Further detail information is still required about the revealed AM fungal population, and then there is possibility to improve better seedling production in nursery and survival rates of *V. pachyceras* seedlings when planted in desert conditions by increasing population of certain AM fungal species in roots and rhizosphere.

## Conclusion

In conclusion, despite harsh climatic conditions prevailing in Kuwait desert, our results demonstrate surprisingly a diverse number of general fungal and AM fungal resources exists in the studied area that remain to be fully characterized. Our study from both rhizospheric and bulk soil revealed four major fungal phyla and classified as Ascomycota, Basidiomycota, Zygomycota and Chytridiomycota. In which Ascomycota is the most abundant phylum (48%) followed by Basidiomycota, Zygomycota and Chytridiomycota. A great number of fungal ITS rDNA sequences were related to a wealth of incertae sedis fungi, suggesting further works are needed to classify them at the genus level. Similar to many other studies, we also found distinct fungal composition and diversity between the rhizospheric and non-rhizospheric soils. The most abundant AM fungal group identified was among the family Glomeraceae. Evidently, further detail taxonomic information on is still required in order to performing quantitative comparisons of relative phylotypes obtained in this research. The current research would however, assists set the basis for future research and might be helpful to determine the strategies used by this fungal microbiota in response to hot and dry weather conditions for plant fitness. Furthermore, to exploit the potential for the use of these fungal species as biofertilizer to unfavorable desert conditions, this might lead to improvement in restoration and revegetation strategies for about to extinct LT species in Kuwait. Apparently, this study is a first effort using molecular approach and advances our existing knowledge about the general fungal, and AM fungal communities related with this nationally important unique tree since the tree species is considered endangered in Kuwait.

## Author Contributions

MS and AQ planned and conceptualized the experiments. MS, AQ, AM, and MS conducted the experiments and wrote the manuscript. KD, LC, PN, and NB supervised and reviewed the manuscript. All authors approved the manuscript.

## Conflict of Interest Statement

The authors declare that the research was conducted in the absence of any commercial or financial relationships that could be construed as a potential conflict of interest.

## Footnotes

1 http://www.mbio.ncsu.edu/BioEdit/bioedit.html

2 http://www.ncbi.nlm.nih.gov/BLAST/
